# Sleep disturbance in post COVID-19 conditions: Prevalence and quality of life

**DOI:** 10.3389/fneur.2022.1095606

**Published:** 2023-01-09

**Authors:** Rimawati Tedjasukmana, Astri Budikayanti, Wardah Rahmatul Islamiyah, Anastasia Melissa Ayu Larasati Witjaksono, Manfaluthy Hakim

**Affiliations:** ^1^Faculty of Medicine, Universitas Kristen Krida Wacana, Jakarta, Indonesia; ^2^Department of Neurology, Faculty of Medicine, Universitas Indonesia, Jakarta, Indonesia; ^3^Department of Neurology, Cipto Mangunkusumo General Hospital, Jakarta, Indonesia; ^4^Department of Neurology, Faculty of Medicine, Universitas Airlangga, Surabaya, Indonesia; ^5^Department of Neurology, Dr. Soetomo General Hospital, Surabaya, Indonesia

**Keywords:** post COVID-19 condition, COVID-19, sleep disturbance, prevalence, quality of life

## Abstract

Post COVID-19 conditions are complaints and symptoms in patients with a history of probable or confirmed COVID-19 after 3 months of the onset of COVID-19 and last at least 2 months. About 10–20% of people may experience post COVID-19 conditions, one of which is sleep disturbance. There is a wide range of prevalence of sleep disturbances from 6% to more than 70%. An online survey of the post COVID-19 conditions in various countries showed that 78.58% of subjects had sleep disturbances, including insomnia, sleep-disordered breathing, central disorders of hypersomnolence, circadian rhythm sleep-wake disorders, parasomnias, and sleep-related movement disorders. Sleep disturbance can be found starting from 2 weeks until 48 weeks or more after discharge or after having a negative COVID-19 test results. Women aged < 50 years old with severe COVID-19 infection reported a worse outcome. Several mechanisms may cause sleep disturbance in post COVID-19 condition, namely persistent viral infection and inflammation, immunity dysregulation, and mitochondrial dysfunction. Several studies discovered sleep disturbance was a major problem that affected different domains of QoL in post COVID-19 conditions. Significant correlation was found between several dimensions of SF-36 with moderate-to-severe insomnia in post COVID-19 conditions. Therefore, sleep disturbance is a major problem in post COVID-19 conditions and may affect patients' QoL, and the existence of sleep disturbance should be a concern in post COVID-19 conditions period. Further research is required to determine the prevalence based on agreed definition as well as methods to assess this condition and its impact on QoL.

## 1. Introduction

Some patients who have suffered from Coronavirus Disease 2019 (COVID-19) may experience prolonged complaints and symptoms known as post COVID-19 conditions. Post COVID-19 conditions is a term of complaints and symptoms that have just appeared or continued after 3 months since the clinical onset of COVID-19 and have lasted for at least 2 months, or a history of close and confirmed contact (1, 2) World Health Organization (WHO) data shows the prevalence of post COVID-19 conditions estimated at 10–20%, with sleep disturbances among the six most complained of disorders in this condition (1). There is a wide range of prevalence of sleep disorders among different countries. Furthermore, there was a study conducted online with respondents from several countries found that 78.6% of patients with post COVID-19 conditions experienced sleep disturbance (2).

The biological process of sleep is crucial for preserving internal balance and overall well-being. In post COVID-19 conditions, several mechanisms, including persistent viral infection, persistent inflammation, involvement of the autoimmune system, and mitochondrial dysfunction can interfere with the part of the brain that regulates the sleep-wake cycle, causing sleep disturbance (3). On top of that, if sleep disturbance is not adequately managed, it can interfere with immune system and will further affect the sleep-wake cycle, which will continue creating a vicious circle and decrease overall QoL.

During the post COVID-19 condition period, sleep disturbance have a significant negative impact on variety of QoL aspects (4). A study conducted by Sigfrid et al. showed that patients with post COVID-19 conditions have a significant 10% decrease in QoL (5). According to Davis et al., 45.2% of the patients with post COVID-19 conditions needed to work fewer hours than they did before getting sick, and a further 22.3% were off work at the time of the survey because of the sickness (2). The Short Form-36 (SF-36)'s several dimensions of QoL had a statistically significant positive association with Insomnia Severity Index (ISI) scale. In addition, Global Pittsburgh Sleep Quality Index (Global PSQI) and mean duration of post COVID-19 conditions also showed a statistically significant positive correlation with various domains of the SF-36 QoL scale (4).

Understanding QoL is crucial for enhancing patient care, symptom alleviation, and rehabilitation. Patients' self-reported QoL issues may prompt therapy and care revisions and improvements, or may demonstrate that some therapies are ineffective. Identifying the variety of issues that may affect patients also involves using QoL (6). Therefore, in this review, we discuss the currently available published literature related to sleep disturbance in post COVID-19 conditions, the prevalence, the possible mechanism, and its association with QoL.

## 2. The post COVID-19 conditions definition

Since the global outbreak of novel coronavirus pneumonia in 2019, known as Coronavirus Disease (COVID-19), the knowledge about the long-term effects of the disease has rapidly grown. There are several definitions and terms for long-term symptomatic COVID-19. This condition was described for the first time by Greenhalgh et al. as post-acute COVID-19 and chronic COVID-19, with post-acute COVID-19 lasting more than 3 weeks after the start of the first symptoms and chronic COVID-19 lasting more than 12 weeks (7). According to the Scottish Intercollegiate Guidelines Network, the Royal College of General Practitioners, and the National Institute for Health and Care Excellence (NICE), long COVID is defined as signs and symptoms that developed during or after a disease that is consistent with COVID-19 and that persist for more than 4 weeks but cannot be accounted for by other diagnoses (8). Therefore, WHO stated that post COVID-19 conditions are complaints and symptoms that occur in patients with a history of probable or confirmed COVID-19 after 3 months of the onset of infection and last at least 2 months (1). These complaints and symptoms of the post COVID-19 conditions cannot be explained by other alternative diagnoses, and have an impact on the patient's daily functioning. Symptoms may persist after infection or appear after a period of recovery. Symptoms can also fluctuate (change in quality and quantity over time) or recur (recurrence of disease manifestations after symptom improvement) (1, 2).

Multiple organs may be affected by the post COVID-19 conditions (2). According to WHO, the most common symptoms and complaints were fatigue (78%), shortness of breath (78%), and cognitive dysfunction (74%). In addition, other symptoms that are often complained of are memory disturbances (65%), muscle pain or spasms (64%), and sleep disturbances (62%) (1, 2). A significant increase of psychological manifestation such as anxiety and depression was noticeably reported in post COVID-19 conditions (9, 10). Noticeably, a meta-analysis study found fatigue, cognitive dysfunction, and sleep disturbance appeared to be the key features of post COVID-19 conditions (1, 9).

## 3. Discussion

### 3.1. Prevalence and types of sleep disturbance in post COVID-19 conditions

Globally, more than 160 million cases of COVID-19 have been confirmed and there have been more than 3 million deaths. The majority of COVID-19 patients have mild to moderate symptoms, whereas 14% of patients have severe symptoms and 5% are in serious condition (11). The median time to recover from COVID-19 is 13 days, with an interquartile range of 9–17 days (12). Despite already having negative PCR test result, 1 in 5 people may have symptoms for 5 weeks or more, while 1 in 10 may be symptomatic for 12 weeks or more (13). In a study conducted by Whitaker et al., 37.7% of 76,155 people with post COVID-19 conditions experienced at least one persistent symptom, while 17.47% experienced three or more symptoms, lasting up to 12 weeks (14). This study also examined the prevalence of post COVID-19 conditions after a period of 12 weeks, they found 5.80% patients with one or more symptoms and 2.23% of patients with three or more symptoms (14). The prevalence of persistent symptoms was higher in women than in men and the risk of post COVID-19 conditions symptoms increased linearly with age (14, 15).

Sleep disturbance were among post COVID-19 conditions that were frequently recorded throughout the pandemic, both during the acute phase of COVID-19 and after recovery, which resulted in ongoing problems for the survivors' lives (16). Rousseau et al. conducted a follow-up study of subjects with severe COVID-19 who were hospitalized for more than 7 days and found that 75% had poor sleep quality. They suggested that the majority of severe COVID-19 survivors have sleep fragmentation and frequent arousals from sleep (17). Davis et al. conducted an online survey of the post COVID-19 conditions phenomenon in various countries. The study results showed that 78.58% of subjects had sleep disturbances (2). Another study conducted by Sigfrid et al. in the UK found that 46.2% of patients experienced sleep disturbances 3 months after initial COVID-19 symptom onset (5).

Studies in North-Africa and the Middle-East have found similar results. 35% of post COVID-19 conditions patients were still displaying sleep disturbance 2 months after discharge from a Bangladesh hospital, primarily in patients with diabetes mellitus (16). A cross-sectional observational study in the Saudi Arabia investigated the sleep disturbances in 32% patient and showed high scores of ISI and PSQI (4). According to a study conducted in Egypt with 85 recovered COVID-19 patients and 85 individuals without COVID-19, most recovered COVID-19 patients (77%) showed sleep disturbance, compared to 46% of controls (18).

Despite the fact that patients with post COVID-19 conditions frequently complain about sleep, but comprehensive data are still lacking. Data from several studies showed that the prevalence of sleep disturbance in post COVID-19 conditions has a wide range between 6% to more than 70% (Table 1). This wide range can be due to differences in methodology used in previous prevalence studies. Some studies included all patients with post COVID-19 conditions, while others only included patients who had sleep disturbances during the acute infection period and were observed throughout their recovery time. Most of the studies in Table 1 mention the types of sleep disturbance experienced by patients in the form of insomnia, while only two studies focused on Obstructive Sleep Apnea (OSA). There was only one study described several types of sleep disturbance and their prevalence, with insomnia at 60%, waking up several times at night at 41%, awakening due to breathing difficulty at 36%, having restless legs syndrome (RLS) at 18%, sleep apnea at 10%, having vivid dreams at 33%, nightmares 26%, and lucid dreams 15% (2). Another cause for this wide range of prevalence might be due to the variations in evaluation time following the onset of COVID-19 in the studies shown in Table 1, starting from 2 weeks until 48 weeks or more after discharge or after having a negative COVID-19 test results (19, 27).

**Table 1:** Type and prevalence of sleep disturbance.

**No**	**Reference**	**Type of sleep disturbance**	**Evaluation time after onset of COVID-19**	**Prevalence**
1.	Xu et al. (19)	Insomnia	2 weeks	26.45% of 121
2.	Zhang et al. (20)	Poor sleep (unspecified)	3 weeks	55.6% of 135
3.	Huynh et al. (21)	Insomnia	2–4 weeks	34.5% of 325
4.	Lorenzo et al. (22)	Insomnia	3–4 weeks	27.6% of 195
5.	Mazza et al. (23)	Insomnia	4 weeks	40% of 402
6.	Ng et al. (24)	Sleep disturbance (unspecified)	4 weeks	77.7% of 18
7.	Halpin et al. (25)	Obstructive sleep apnea	4–8 weeks	15.3% of 100
8.	Kalamara et al. (26)	Insomnia	4 weeks 12 weeks 24 weeks	56.5% 53.5% 39.2%
9.	Buensenso et al. (27)	Insomnia	4–20 weeks 24–36 weeks ≥48 weeks	9.3% of 355 5.7% of 157 5.2% of 154
10.	Islam et al. (16)	Sleep disturbance (unspecified)	8 weeks	35% of 322
11.	Arnold et al. (28)	Insomnia	8–12 weeks	24% of 110
12.	Pellitteri et al. (29)	Insomnia	8 weeks 40 weeks	19.1% of 47 27.3% of 44
13.	Magdy et al. (30)	Insomnia	12 weeks	Migraine patient: 23.5% of 204 Non-migraine patient: 12.3% of 204
14.	Moy et al. (31)	Insomnia	>12 weeks >24 weeks 48 weeks	8 of 126 19% of 60 45% of 60
15.	Alkodaymi et al. (32)	Sleep disorder (unspecified)	12–24 weeks 24–36 weeks 36–48 weeks >48 weeks	24% of 257,248 29% of 257,248 0% 30% of 257,248
16.	Sayed et al. (4)	Insomnia	16 weeks	32% of 500
17.	Mattioli et al. (33)	Insomnia	16 weeks	6.6% of 120
18.	Labarca et al. (34)	Obstructive sleep apnea	16 weeks	61.6% of 60
19.	Taquet et al. (35)	Insomnia	24 weeks	5.42% of 236,379
21.	Huang et al. (36)	Sleep disturbance (unspecified)	24 weeks	26% of 1,655
22.	Elkan et al. (37)	Sleep disturbance (unspecified)	36 weeks	8% of 66
23.	Davis et al. (2)	Insomnia Night sweats Awakened feeling unable to breathe Restless legs Sleep apnea Vivid dreams Nightmares Lucid dreams	N/A	60% 41% 36% 18% 10% 33% 26% 15%

### 3.2. The possible mechanism of sleep disturbance in post COVID-19 conditions

Although no specific pathophysiological theory can define post COVID-19 conditions, attention has been directed toward several mechanisms, including persistent viral infection, persistent inflammation, involvement of the autoimmune system, and mitochondrial dysfunction (3). One of the mechanisms, namely persistent viral infection indicated by persisting SARS-CoV-2 RNA for up to 230 days after the onset of symptoms in several anatomic areas, including the brain's various regions (38). Since the virus also affects the hypothalamus and brainstem, it may disrupt the sleep-wake cycle, resulting in insomnia or poor quality of sleep (39).

Persistent proinflammatory cells [IL-1, IL-6, and Tumor Necrosis Factor-α (TNF-α)] and altered cytokine production is suspected to have occurred during persistent inflammation in post COVID-19 conditions, revealed by a cytokine profiling study (40, 41). Inflammation can modify sleep by increasing Non-Rapid Eye Movement (NREM) sleep and decreasing rapid eye movement (REM) sleep, and vice versa sleep disturbance can also modify the inflammation process by altering circulating cytokines (41, 42).

There are evidence pointing that post COVID-19 conditions involves autoimmunity (43). Dysregulation of this autoimmune response may cause sleep dysfunction. Both humoral and cellular immune-mediated response can target sleep-regulating neural structures (e.g., brainstem, hypothalamus) as well as neurotransmitter systems (e.g., hypocretin) (40, 44).

**Figure 1:**
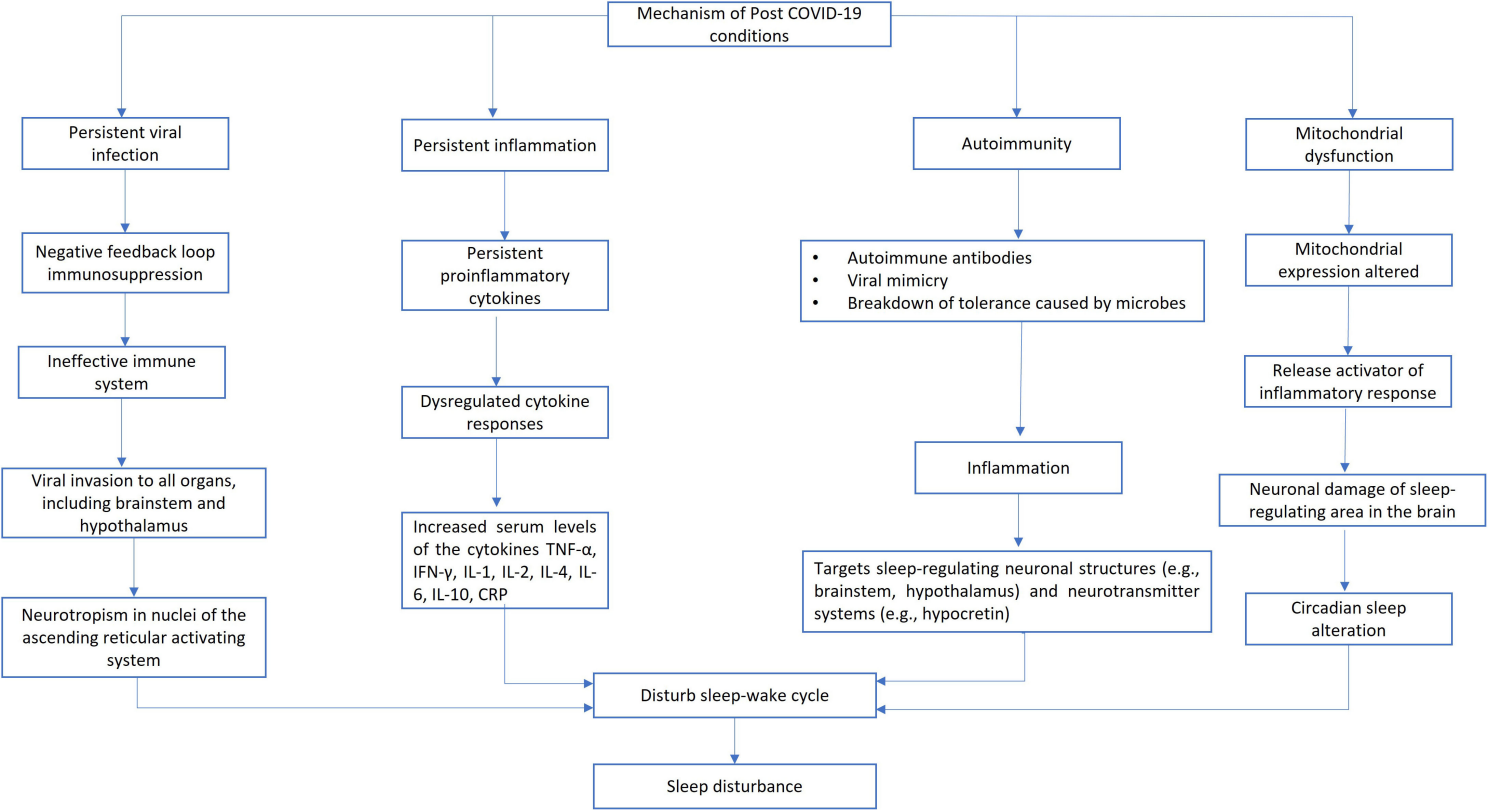
Possible pathogenic mechanisms of sleep disturbance in post COVID-19 conditions.

Mitochondrial dysfunction in post COVID-19 conditions was reported in a small cohort that discovered lower fatty acid oxidation and elevated lactate levels early in workout, showing that mitochondrial dysfunction was present and metabolic reprogramming had occurred (45). Damaged mitochondria can release a wide range of damage-associated molecular patterns that are potent activators of the inflammatory response (46). This process can damage the neurons in the sleep-regulating area of the brain, which affects the circadian rhythm, sleep apnea, and obstructive sleep apnea (47, 48). Figure 1 summarized all possible pathogenic mechanisms of sleep disturbance in post COVID-19 conditions.

### 3.3. Factors contributing to sleep disturbance in post COVID-19 conditions

Several factors may contribute to sleep disturbances in post COVID-19 conditions. Disease severity, circadian rhythm disturbance, psychiatric disturbance, uncontrolled chronic diseases during acute stage, social isolation, social economic status, age and even gender may play a role. When individuals with moderately severe symptoms are treated in hospitals, hospitalization might alter the circadian rhythm, which may lead patients to have sleep disturbances. Circadian rhythms, which are primarily triggered by daylight, meals, and exercise (49) are frequently compromised or altered while a patient is hospitalized (50). COVID-19 patients tend to experience fear and anxiety about social isolation procedures, as well as mandatory lockdowns in the acute phase, leading to an increased incidence of sleep disturbances in the population (51–53). A study reported a higher prevalence of difficulty falling asleep, staying asleep, and getting up early in patients with moderately severe COVID-19 infection, whereas difficulty staying asleep and waking up earlier was experienced in patients with more severe disease (54). Another study showing that diabetes mellitus was independently associated with sleep disturbances among patients with post COVID-19 conditions showed that the presence of chronic illnesses can also have an impact on the persistence of sleep disturbance (16). COVID-19 patients conveyed that the disturbing factors that affect them are a sense of tightness, worries about illness, anxiety, the voices of other patients, medical staff, and medical devices (50). Another study using actigraphy found that patients with severe COVID-19 symptoms, including those with severe respiratory symptoms and those requiring Intensive Care Unit (ICU) displayed lower sleep efficiency, higher immobility time, and higher fragmentation index compared to those with mild symptoms and who do not require ICU care (55). Actigraphy may not be the gold standard for evaluating sleep, but it is simple to implement and could be sufficient for identifying changes in sleep schedules that would indicate disturbances of the circadian clock (56).

There is a bidirectional relationship between sleep disturbance and psychiatric disorders (57). A study described that patients with post COVID-19 condition who listed mental health issues such as stress, anxiety, or depression were more likely to develop sleep disturbance compared to those who did not (21). Another study reported that moderate to severe sleep disturbances associated with mood symptoms evaluated using the Patient Health Questionnaire (PHQ)-2 and the Anxiety Disorder (GAD)-2 questionnaires (58). It is critical that healthcare professionals evaluate and treat sleep disturbance among patients with post COVID-19 conditions in order to improve both their regular sleep patterns and mental health because adequate sleep is essential for facilitating patient recovery (21).

A study evaluated insomnia in post COVID-19 conditions reported that age and health status may be the influencing factors. Insomnia is more common in older individuals because they tend to experience a low self-evaluation and are more prone to be under multiple strains, including social economy and health-related problem (19). Among patients with post COVID-19 conditions, another study reported that women also predicted poor result of global PSQI (18). According to a study, women are more likely than men to survive severe acute illness, which may lead to worse long-term results. However, more investigation is required to demonstrate why women predominately experience the post COVID-19 conditions (5).

### 3.4. Quality of life of sleep disturbance in post COVID-19 conditions

The Quality of Life (QoL) was defined as “a patient's general subjective perception of the burden of sickness or medical condition on various areas, including physical, psychological, social, and occupational functioning” (6). The QoL of patients with post COVID-19 conditions has been significantly affected regardless of the period of time after discharge or recovery. A study conducted by Sigfrid et al. reported that patients with post COVID-19 conditions showed a significant 10% decrease in QoL (5). This measurement is based on 5 dimensions, consisting of mobility, self-care, activities of daily living, pain or discomfort, and anxiety/depression (5, 59). The most frequently discussed risk factors for poor QoL included female sex, advanced age, co-morbidities, Intensive Care Unit (ICU) admission, prolonged ICU stay, and mechanical ventilation (60).

There was still a significant persisting impact on QoL across all dimensions in patients with post COVID-19 conditions 12 weeks after onset in both survivors and family members (61). During the period of post COVID-19 conditions, sleep issues must be handled because they have severe effects on numerous areas of QoL. The SF-36's several dimensions of QoL had a statistically significant positive association with moderate-to-severe insomnia based on ISI scale. One third of total patient had moderate-to-severe insomnia showed significant positive association with several dimensions of SF-36, such as physical functioning, role limitation due to physical health, role limitation due to emotional problems, and general health. Global PSQI score of post COVID-19 conditions also showed a statistically significant positive correlation with various domains of the SF-36 QoL scale (4). Another study also indicated that poor sleepers based on PSQI examination significantly underperformed in several SF-36 areas relating to subjective physical and mental health indicators when compared to good ones (29).

There was a high proportion of patients with post COVID-19 conditions who also suffered from mental health issues such as stress, anxiety, and depression (21). A study using Short Form-8 (SF-8) to measure QoL found that post-Traumatic Stress Disorder (PTSD) has mediating effect on insomnia and QoL (62). Since sleep disturbances and mental health are strongly associated, intervention and prevention strategies concerning mental health issues may improve Health-Related Quality of Life (HRQoL) and sleep in patients with post COVID-19 conditions (62).

Regardless of the amount of time after discharge or recuperation, the QoL of the patients with post COVID-19 conditions was significantly impacted (60). Understanding QoL is crucial for enhancing patient care, symptom alleviation, and rehabilitation. Patients' self-reported QoL issues may prompt therapy and care revisions and improvements, or they may demonstrate that some therapies are ineffective (6). Therefore, it is important to take further preventive steps in order to stop the sleep disturbance in patients with post COVID-19 conditions from worsening and affecting QoL.

## 4. Conclusion

Sleep disturbances have a strong impact in decreasing post COVID-19 conditions' overall QoL. Various kinds of sleep disturbance can be found in patients with post COVID-19 conditions with a wide range of prevalence, between 6% to more than 70%. This wide range may be caused by differences in methodology and timing of assessments following the onset of COVID-19. No specific pathophysiological theory can define the post COVID-19 conditions or sleep disturbance in post COVID-19 conditions. Still, several mechanisms that play a role in post COVID-19 conditions can cause sleep disturbance, namely persistent viral infection, persistent inflammation, autoimmunity or immunity dysregulation, and mitochondrial dysfunction. In addition, other factors such as disease severity, circadian rhythm disturbance, psychiatric disturbance, uncontrolled chronic diseases during acute stage, social isolation, social economic status, age, and even gender may also play a role. Sleep disturbance positively correlates with several dimensions SF-36, a measurement tool for QoL. By better understanding the impact of sleep disturbance on QoL, the existence of sleep disturbance should be a concern in post COVID-19 conditions period. Besides, it is important to take further preventive steps in order to stop the sleep disturbance in post COVID-19 conditions from worsening and affecting QoL. Further research is required to determine the natural history of this condition as well as to define risk factors, pathogenesis, the prevalence in various countries, and therefore determine possible interventional strategies.

## Author contributions

All authors participating in conceptual framework, study design, literature review, and drafting the manuscript for publication.

## References

[1] World Health Organization. A Clinical Case Definition of Post COVID-19 Condition by a Delphi Consensus Data. (2021). Available online at: WHO/2019-nCoV/Post_COVID-19_condition/Clinical_case_definition/20211 (accessed on: December 23, 2021).

[2] Davis HE Assaf GS McCorkell L Wei H Low RJ Re'em Y. Characterizing long COVID in an international cohort: 7 months of symptoms and their impact. eClinicalMedicine. (2021) 38:101019. 10.1016/j.eclinm.2021.101019 34308300 PMC8280690

[3] Watanabe H Shima S Mizutani Y Ueda A Ito M [Long COVID: Pathogenesis and Therapeutic Approach]. Brain Nerve. (2022) 74:879–84. 10.11477/mf.1416202142 35860935

[4] El Sayed S Gomaa S Shokry D Kabil A Eissa A. Sleep in post-COVID-19 recovery period and its impact on different domains of quality of life. Egypt J Neurol Psychiatry Neurosurg. (2021) 57:172. 10.1186/s41983-021-00429-7 34924750 PMC8669420

[5] Sigfrid L Drake TM Pauley E Jesudason EC Olliaro P Lim WS. Long Covid in adults discharged from UK hospitals after Covid-19: a prospective, multicentre cohort study using the ISARIC WHO clinical characterisation protocol. Lancet Reg Heal - Eur. (2021) 8:100186. 10.1016/j.lanepe.2021.100186 34386785 PMC8343377

[6] Haraldstad K Wahl A Andenæs R Andersen JR Andersen MH Beisland E. A systematic review of quality of life research in medicine and health sciences. Qual Life Res. (2019) 28:2641. 10.1007/s11136-019-02214-9 31187410 PMC6761255

[7] Greenhalgh T Sivan M Delaney B Evans R Milne R. Long covid—an update for primary care. BMJ. (2022) 378:e072117. 10.1136/bmj-2022-072117 36137612

[8] National Institute for Health Care Excellence (NICE). Covid19 Rapid Guideline: Managing the Longterm Effects of COVID-19. (2022). Available online at: https://www.nice.org.uk/guidance/ng188/resources/covid19-rapid-guideline-managing-the-longterm-effects-of-covid19-pdf-51035515742 (accessed September 30, 2022).

[9] Premraj L Kannapadi NV Briggs J Seal SM Battaglini D Fanning J. Mid and long-term neurological and neuropsychiatric manifestations of post-COVID-19 syndrome: a meta-analysis. J Neurol Sci. (2022) 434:120162. 10.1016/j.jns.2022.120162 35121209 PMC8798975

[10] Vanichkachorn G Newcomb R Cowl CT Murad MH Breeher L Miller S. Post-COVID-19 syndrome (long haul syndrome): description of a multidisciplinary clinic at mayo clinic and characteristics of the initial patient cohort. Mayo Clin Proc. (2021) 96:1782–91. 10.1016/j.mayocp.2021.04.024 34218857 PMC8112396

[11] Wu Z McGoogan JM. Characteristics of and important lessons from the coronavirus disease 2019 (COVID-19) Outbreak in China: Summary of a Report of 72 314 Cases From the Chinese Center for Disease Control and Prevention. JAMA. (2020) 323:1239–42. 10.1001/jama.2020.2648 32091533

[12] Kaso AW Hareru HE Kaso T Agero G. Time to recovery from Covid-19 and its associated factors among patients hospitalized to the treatment center in South Central Ethiopia. Environ Challenges. (2022) 6:100428. 10.1016/j.envc.2021.100428 PMC8673952 36632239

[13] Office for National Statistics. The prevalence of long COVID symptoms and COVID-19 complications - Office for National Statistics. Office for National Statistics (ONS) (2020). Available online at: https://www.ons.gov.uk/news/statementsandletters/theprevalenceoflongcovid symptomsandcovid19complications (accessed October 13, 2022).

[14] Whitaker M Elliott J Chadeau-Hyam M Riley S Darzi A Cooke G. Persistent COVID-19 symptoms in a community study of 606,434 people in England. Nat Commun. (2022) 13:1–10. 10.1038/s41467-022-29521-z 35413949 PMC9005552

[15] Sudre CH Murray B Varsavsky T Graham MS Penfold RS Bowyer RC. Attributes and predictors of long COVID. Nat Med. (2021) 27:626. 10.1038/s41591-021-01292-y 33692530 PMC7611399

[16] Islam MK Molla MMA Hasan P Sharif MM Hossain FS Amin MR. Persistence of sleep disturbance among post-COVID patients: Findings from a 2-month follow-up study in a Bangladeshi cohort. J Med Virol. (2022) 94:971–8. 10.1002/jmv.27397 34647638

[17] Rousseau AF Minguet P Colson C Kellens I Chaabane S Delanaye P. Post-intensive care syndrome after a critical COVID-19: cohort study from a Belgian follow-up clinic. Ann Intens Care. (2021) 11:118. 10.1186/s13613-021-00910-9 34324073 PMC8319705

[18] Abdelghani M Alsadik ME Abdelmoaty AA Atwa SA Said A Hassan MS. WHO EMRO | Sleep disturbances following recovery from COVID-19: a comparative cross-sectional study, Egypt | Research articles. East Mediterr Health J. (2022) 28:14–22. 10.26719/emhj.22.006 35165874

[19] Xu F Wang X Yang Y Zhang K Shi Y Xia L. Depression and insomnia in COVID-19 survivors: a cross-sectional survey from Chinese rehabilitation centers in Anhui province. Sleep Med. (2022) 91:161–5. 10.1016/j.sleep.2021.02.002 33627300 PMC7869685

[20] Zhang J Xu D Xie B Zhang Y Huang H Liu H. Poor-sleep is associated with slow recovery from lymphopenia and an increased need for ICU care in hospitalized patients with COVID-19: a retrospective cohort study. Brain Behav Immun. (2020) 88:50. 10.1016/j.bbi.2020.05.075 32512133 PMC7274970

[21] Huynh G Nguyen HV Vo LY Le NT Nguyen HTN. Assessment of insomnia and associated factors among patients who have recovered from COVID-19 in Vietnam. Patient Prefer Adherence. (2022) 16:1637–47. 10.2147/PPA.S371563 35837086 PMC9275485

[22] De Lorenzo R Conte C Lanzani C Benedetti F Roveri L Mazza MG. Residual clinical damage after COVID-19: a retrospective and prospective observational cohort study. PLoS One. (2020) 15:e0239570. 10.1371/journal.pone.0239570 33052920 PMC7556454

[23] Mazza MG De Lorenzo R Conte C Poletti S Vai B Bollettini I. Anxiety and depression in COVID-19 survivors: role of inflammatory and clinical predictors. Brain Behav Immun. (2020) 89:594–600. 10.1016/j.bbi.2020.07.037 32738287 PMC7390748

[24] Ng R Vargas G Jashar DT Morrow A Malone LA. Neurocognitive and psychosocial characteristics of pediatric patients with post-acute/long-COVID: a retrospective clinical case series. Arch Clin Neuropsychol. (2022) 37:1633–43. 10.1093/arclin/acac056 35901463 PMC9384547

[25] Halpin SJ McIvor C Whyatt G Adams A Harvey O McLean L. Postdischarge symptoms and rehabilitation needs in survivors of COVID-19 infection: a cross-sectional evaluation. J Med Virol. (2021) 93:1013–22. 10.1002/jmv.26368 32729939

[26] Kalamara E Pataka A Boutou A Panagiotidou E Georgopoulou A Ballas E. Persistent Sleep Quality Deterioration among Post-COVID-19 Patients: Results from a 6-Month Follow-Up Study. J Pers Med. (2022) 12:1909. 10.3390/jpm12111909 36422085 PMC9692708

[27] Buonsenso D Pazukhina E Gentili C Vetrugno L Morello R Zona M. The prevalence, characteristics and risk factors of persistent symptoms in non-hospitalized and hospitalized children with SARS-CoV-2 infection followed-up for up to 12 months: a prospective, cohort study in Rome, Italy. J Clin Med. (2022) 11:6772. 10.3390/jcm11226772 36431250 PMC9692851

[28] Arnold DT Hamilton FW Milne A Morley AJ Viner J Attwood M. Patient outcomes after hospitalisation with COVID-19 and implications for follow-up: Results from a prospective UK cohort. Thorax. (2021) 76:399–401. 10.1136/thoraxjnl-2020-216086 33273026 PMC7716340

[29] Pellitteri G Surcinelli A De Martino M Fabris M Janes F Bax F. Sleep alterations following COVID-19 are associated with both neuroinflammation and psychological disorders, although at different times. Front Neurol. (2022) 13:929480. 10.3389/fneur.2022.929480 36062000 PMC9428349

[30] Magdy R Elmazny A Soliman SH Elsebaie EH Ali SH Abdel Fattah AM. Post-COVID-19 neuropsychiatric manifestations among COVID-19 survivors suffering from migraine: a case–control study. J Headache Pain. (2022) 23:101. 10.1186/s10194-022-01468-y 35962348 PMC9372973

[31] Moy FM Hairi NN Lim ERJ Bulgiba A. Long COVID and its associated factors among COVID survivors in the community from a middle-income country—An online cross-sectional study. PLoS ONE. (2022) 17:e0273364. 10.1371/journal.pone.0273364 36040960 PMC9426885

[32] Alkodaymi MS Omrani OA Fawzy NA Shaar BA Almamlouk R Riaz M. Prevalence of post-acute COVID-19 syndrome symptoms at different follow-up periods: a systematic review and meta-analysis. Clin Microbiol Infect. (2022) 28:657. 10.1016/j.cmi.2022.01.014 35124265 PMC8812092

[33] Mattioli F Stampatori C Righetti F Sala E Tomasi C De Palma G. Neurological and cognitive sequelae of Covid-19: a four month follow-up. J Neurol. (2021) 268:4422. 10.1007/s00415-021-10579-6 33932157 PMC8088203

[34] Labarca G Henríquez-Beltrán M Lamperti L Nova-Lamperti E Sanhueza S Cabrera C. Impact of Obstructive Sleep Apnea (OSA) in COVID-19 Survivors, Symptoms Changes Between 4-Months and 1 Year After the COVID-19 Infection. Front Med. (2022) 9:884218. 10.3389/fmed.2022.884218 35775008 PMC9237467

[35] Taquet M Geddes JR Husain M Luciano S Harrison PJ. 6-month neurological and psychiatric outcomes in 236 379 survivors of COVID-19: a retrospective cohort study using electronic health records. The Lancet Psychiatry. (2021) 8:416–27. 10.1016/S2215-0366(21)00084-5 33836148 PMC8023694

[36] Huang C Huang L Wang Y Li X Ren L Gu X. 6-month consequences of COVID-19 in patients discharged from hospital: a cohort study. Lancet (London, England). (2021) 397:220. 10.1016/S0140-6736(20)32656-8 33428867 PMC7833295

[37] Elkan M Dvir A Zaidenstein R Keller M Kagansky D Hochman C. Patient-reported outcome measures after hospitalization during the COVID-19 pandemic: a survey among COVID-19 and non-COVID-19 patients. Int J Gen Med. (2021) 14:4829. 10.2147/IJGM.S323316 34471377 PMC8405220

[38] Chertow DS Stein SR Ramelli SC Grazioli A Chung JY Singh M. SARS-CoV-2 infection and persistence throughout the human body and brain. Nat Portf . (2022). Available online at: https://assets.researchsquare.com/files/rs-1139035/v1_covered.pdf?c=1640020576 (accessed October 30, 2022).

[39] Gupta R Pandi-Perumal SR. SARS-CoV-2 Infection: Paving Way for Sleep Disorders in Long Term! Sleep Vigil. (2021) 5:1. 10.1007/s41782-021-00145-5 34027301 PMC8126505

[40] Mehandru S Merad M. Pathological sequelae of long-haul COVID. Nat Immunol 2022 232. (2022) 23:194–202. 10.1038/s41590-021-01104-y 35105985 PMC9127978

[41] Schultheiß C Willscher E Paschold L Gottschick C Klee B Henkes S-S. The IL-1β, IL-6, and TNF cytokine triad is associated with post-acute sequelae of COVID-19. Cell Reports Med. (2022) 3:100663. 10.1016/j.xcrm.2022.100663 35732153 PMC9214726

[42] Hurtado-Alvarado G Pavón L Castillo-García SA Hernández ME Domínguez-Salazar E Velázquez-Moctezuma J. Sleep loss as a factor to induce cellular and molecular inflammatory variations. Clin Dev Immunol. (2013) 2013:801341. 10.1155/2013/801341 24367384 PMC3866883

[43] Cañas CA. The triggering of post-COVID-19 autoimmunity phenomena could be associated with both transient immunosuppression and an inappropriate form of immune reconstitution in susceptible individuals. Med Hypotheses. (2020) 145:110345. 10.1016/j.mehy.2020.110345 33080459 PMC7556280

[44] Iranzo A. Sleep and neurological autoimmune diseases. Neuropsychopharmacology. (2020) 45:129. 10.1038/s41386-019-0463-z 31302665 PMC6879573

[45] de Boer E Petrache I Goldstein NM Olin JT Keith RC Modena B. Decreased fatty acid oxidation and altered lactate production during exercise in patients with post-acute COVID-19 syndrome. Am J Respir Crit Care Med. (2022) 205:126–9. 10.1164/rccm.202108-1903LE 34665688 PMC8865580

[46] Casaril AM Dantzer R Bas-Orth C. Neuronal mitochondrial dysfunction and bioenergetic failure in inflammation-associated depression. Front Neurosci. (2021) 15:1472. 10.3389/fnins.2021.725547 34790089 PMC8592286

[47] Palagini L Geoffroy PA Miniati M Perugi G Biggio G Marazziti D. Insomnia, sleep loss, and circadian sleep disturbances in mood disorders: a pathway toward neurodegeneration and neuroprogression? A theoretical review*.CNS Spectr*. (2022) 27:298–308. 10.1017/S1092852921000018 33427150

[48] Ramezani RJ Stacpoole PW. Sleep disorders associated with primary mitochondrial diseases. J Clin Sleep Med. (2014) 10:1233–9. 10.5664/jcsm.4212 25325607 PMC4224726

[49] Tahara Y Shibata S. Entrainment of the mouse circadian clock: Effects of stress, exercise, and nutrition. Free Radic Biol Med. (2018) 119:129–38. 10.1016/j.freeradbiomed.2017.12.026 29277444

[50] van den Ende ES van Veldhuizen KDI Toussaint B Merten H van de Ven PM Kok NA. Hospitalized COVID-19 Patients Were Five Times More Likely to Suffer From Total Sleep Deprivation Compared to Non-COVID-19 Patients an observational comparative study. Front Neurosci. (2021) 15:1263. 10.3389/fnins.2021.680932 34675762 PMC8525610

[51] Jahrami H BaHammam AS Bragazzi NL Saif Z Faris M Vitiello M V. Sleep problems during the COVID-19 pandemic by population: a systematic review and meta-analysis. J Clin Sleep Med. (2021) 17:299. 10.5664/jcsm.8930 33108269 PMC7853219

[52] Xiang Y-T Yang Y Li W Zhang L Zhang Q Cheung T. Timely mental health care for the 2019 novel coronavirus outbreak is urgently needed. Lancet Psychiatry. (2020) 7:228–9. 10.1016/S2215-0366(20)30046-8 32032543 PMC7128153

[53] Lin CY Broström A Griffiths MD Pakpour AH. Investigating mediated effects of fear of COVID-19 and COVID-19 misunderstanding in the association between problematic social media use, psychological distress, and insomnia. Internet Interv. (2020) 21:100345. 10.1016/j.invent.2020.100345 32868992 PMC7449889

[54] Henríquez-Beltrán M Labarca G Cigarroa I Enos D Lastra J Nova-Lamperti E. Sleep health and the circadian rest-activity pattern four months after COVID-19. J Bras Pneumol. (2022) 48:e20210398. 10.36416/1806-3756/e20210398 35508066 PMC9064633

[55] Vitale JA Perazzo P Silingardi M Biffi M Banfi G Negrini F. Is disruption of sleep quality a consequence of severe Covid-19 infection? A case-series examination. (2020) 37:1110–4. 10.1080/07420528.2020.1775241 32573293

[56] Herz RS Herzog ED Merrow M Noya SB. The Circadian Clock, the Brain, and COVID-19: The Cases of Olfaction and the Timing of Sleep. J Biol Rhythms. (2021) 36:423–31. 10.1177/07487304211031206 34396817 PMC8442129

[57] Sun X Liu B Liu S Wu DJH Wang J Qian Y. Sleep disturbance and psychiatric disorders: a bidirectional Mendelian randomisation study. Epidemiol Psychiatr Sci. (2022) 31:e26. 10.1017/S2045796021000810 35465862 PMC9069588

[58] Orbea CP Lapin B Katzan I Englund K Foldvary-Schaefer N Mehra R. 0735 Sleep Disturbances in Post-Acute Sequelae of COVID-19 (PASC). Sleep. (2022) 45:A321. 10.1093/sleep/zsac079.731 PMC10072019 37014604

[59] Schilling C Meyer-Lindenberg A Schweiger JI. Cognitive disorders and sleep disturbances in long COVID. Nervenarzt. (2022) 93:779–87. 10.1007/s00115-022-01297-z 35576015 PMC9109661

[60] Nandasena HMRKG Pathirathna ML Atapattu AMMP Prasanga PTS. Quality of life of COVID 19 patients after discharge: Systematic review. PLoS One. (2022) 17:e0263941. 10.1371/journal.pone.0263941 35171956 PMC8849513

[61] Shah R Ali FM Nixon SJ Ingram JR Salek SM Finlay AY. Measuring the impact of COVID-19 on the quality of life of the survivors, partners and family members: a cross-sectional international online survey. BMJ Open. (2021) 11:e047680. 10.1136/bmjopen-2020-047680 34035105 PMC8154981

[62] Mahmoudi H Saffari M Movahedi M Sanaeinasab H Rashidi-Jahan H Pourgholami M. A mediating role for mental health in associations between COVID-19-related self-stigma, PTSD, quality of life, and insomnia among patients recovered from COVID-19. Brain Behav. (2021) 11:e02138. 10.1002/brb3.2138 33811451 PMC8119851

